# Artificial intelligence enhanced ultrasound (AI-US) in a severe obese parturient: a case report

**DOI:** 10.1186/s13089-022-00283-5

**Published:** 2022-08-03

**Authors:** Christian Compagnone, Giulia Borrini, Alberto Calabrese, Mario Taddei, Valentina Bellini, Elena Bignami

**Affiliations:** Anesthesiology, Critical Care, and Pain Medicine Division, Department of Medicine and Surgery, University of Parma, Parma, Italy

**Keywords:** Neuraxial ultrasound, Labor analgesia, Epidural anesthesia, Artificial intelligence, Obesity

## Abstract

**Background:**

Neuraxial anesthesia in obese parturients can be challenging due to anatomical and physiological modifications secondary to pregnancy; this led to growing popularity of spine ultrasound in this population for easing landmark identification and procedure execution.

Integration of Artificial Intelligence with ultrasound (AI-US) for image enhancement and analysis has increased clinicians' ability to localize vertebral structures in patients with challenging anatomical conformation.

**Case presentation:**

We present the case of a parturient with extremely severe obesity, with a Body Mass Index (BMI) = 64.5 kg/m^2^, in which the AI-Enabled Image Recognition allowed a successful placing of an epidural catheter.

**Conclusions:**

Benefits gained from AI-US implementation are multiple: immediate recognition of anatomical structures leads to increased first-attempt success rate, making easier the process of spinal anesthesia execution compared to traditional palpation methods, reducing needle placement time for spinal anesthesia and predicting best needle direction and target structure depth in peridural anesthesia.

## Background

According to the WHO, excess body weight represents one of the most severe public health challenges of the twenty-first century in Europe. This issue affects more women than men and is reflected in a severe increase of obesity in pregnant women, with all the related risks [1].

Neuraxial analgesia is currently the most effective option for pain management during labor. However, in obese parturient, central neuraxial blocks can be challenging due to anatomical and physiological modifications secondary to pregnancy and the underlying disease [2].

In this population, considering the higher frequency of comorbidities and the higher risk of obstetric complications, epidural catheter placement can be a lifeline in an emergent or unplanned conversion to cesarean Sect [3].

To this end, neuraxial ultrasonography has become increasingly popular for epidural space identification. Recently, the integration of Artificial Intelligence for ultrasound image (AI-US) enhancement and analysis has further increased clinicians' ability to locate spine structures in patients with challenging anatomical conformations.

In the present case, a portable handheld AI enhanced ultrasound device played a key role in successfully placing an epidural catheter in a parturient with extreme obesity (BMI = 64.5 kg/m^2^), proving to be superior to palpation and conventional spine ultrasound imaging [4].

## Case presentation

After obtaining written informed consent from the patient for publication of this case report and accompanying images, we present the case of a 37-year-old woman (gravida 2 para 1, gestational age 38 weeks + 5 days) who requested epidural analgesia for labor. The measured patient's height was 153 cm and her weight, on the day before delivery, was 151 kg, with a calculated BMI of 64.5 kg/m^2^. Past medical history was relevant for pharmacologically treated gestational hypothyroidism and diabetes during the previous pregnancy.

**Fig. 1:**
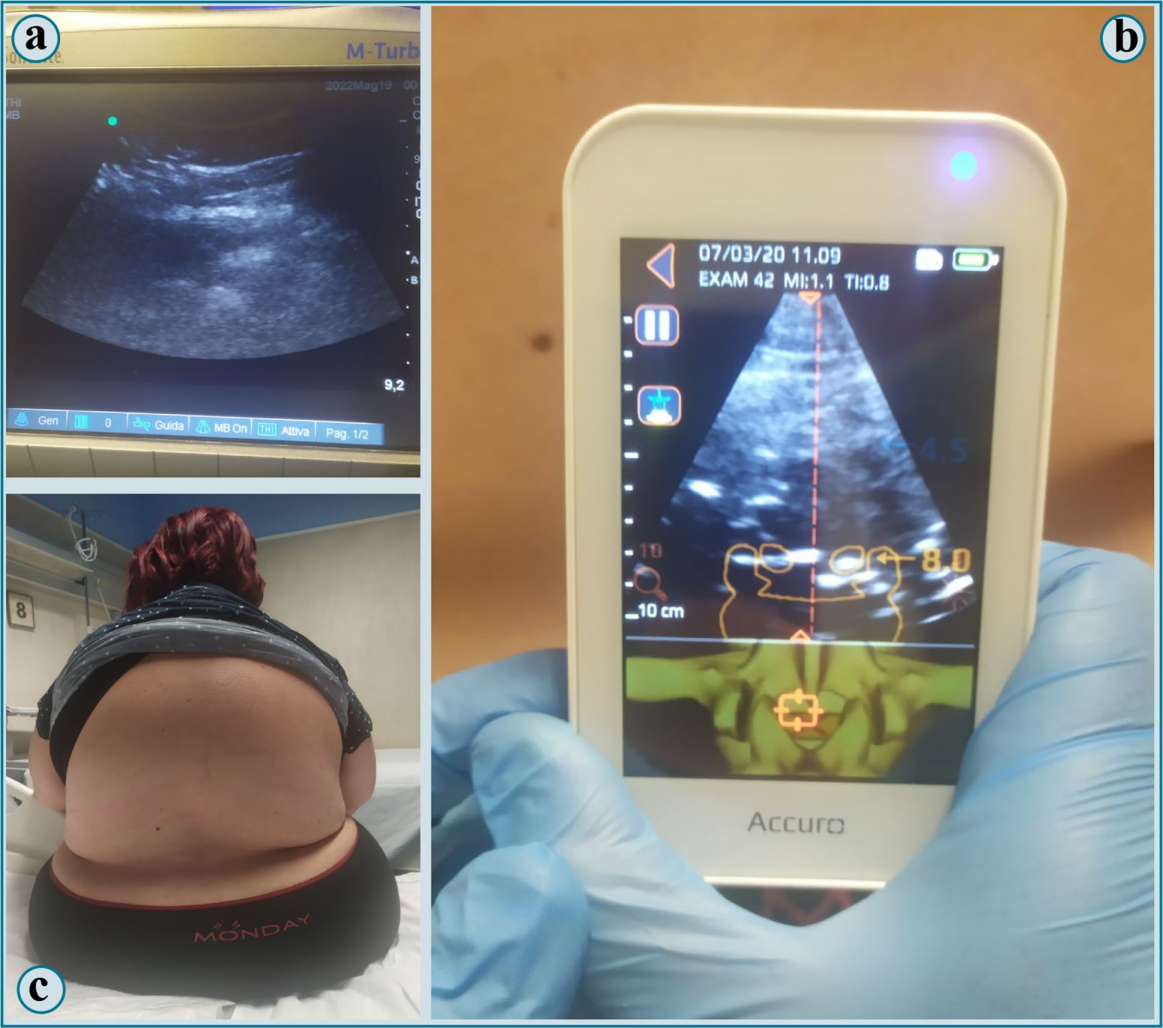
**a** Image obtained with s-US; **b** image obtained with AI-US; **c** surface anatomy of the parturient's back: no anatamical landmark can be identified with palpation

Manual palpation of the spine was carried out with the patient in sitting position; certain localization of interspinous spaces was not possible (Fig. 1, image c).

Lumbar spine ultrasound imaging was then acquired using a standard ultrasound (s-US) machine (SonoSite M-turbo^®^) with a convex probe (Fig. 1, image a). Insonation of the spine was performed by placing the probe midline at sacrum level, in the transverse orientation, and then shifting it cephalad to recognize intervertebral spaces and posterior and anterior complexes. Even with s-US aid, locating interspinous spaces was not feasible, owing to the quantity of subcutaneous tissue that made identifying target structures arduous.

As a last resort, we decided to employ AI-US, a dedicated handheld device combining real-time ultrasound (Fig. 1, image b) with machine learning to assist identification of anatomical structures of the spine (Accuro^®^, Rivanna Medical, Charlottesville, VA, USA). With it, we could identify desired intervertebral space, correct needle insertion point—marked on the patient's skin—and estimated skin-to-epidural space distance—estimated 8 cm, with a slight inclination to the left. It is important to note the inconsistency between the insertion point localized with Accuro^®^ and that presumed after landmark palpation.

The epidural catheter was then placed by a senior attending physician, requiring a single attempt; epidural space was encountered at a 10 cm depth (with a slight discrepancy compared with AI-US esteem—most likely due to tissue compression during images acquisition). Satisfactory labor analgesia was then administered through the epidural catheter. No procedural complications are to be reported.

## Conclusions

We could not find descriptions of epidural catheter placement AI-US assisted in parturient with such high BMI value in the available literature. In the case we present, morphological alterations secondary to pregnancy and obesity created difficulties that could not be overcome using traditional landmark palpation nor standard ultrasound techniques. Nevertheless, the implementation of AI-US has determined the first-step success of the procedure [5].

Preprocedural ultrasound of the patient's lumbar region helps with obtaining important information about spine anatomy: midline identification, optimal vertebral level for catheter placement, the inclination of vertebral bodies and processes and the distance from the skin to the epidural space [6].

Pre-puncture ultrasound is well-known to reduce the number of attempts and significantly increase parturients’ satisfaction in regard to the procedure [7].

This technique is even more helpful when applied in those cases with anticipated difficulty, including anatomical alteration of the lumbar spine and a body mass index (BMI) > 33 kg/m^2^ [8].

However, neuraxial s-US in pregnancy, especially in obese patients, can be tricky as the visibility of the ligamentum flavum, dura mater, and epidural space decreases significantly during pregnancy. In addition, the distance from the skin to the epidural space seems to increase proportionally to BMI [9].

Becoming familiar with the sonoanatomy of the spinal column requires a high level of technical expertise, so that adoption of neuraxial ultrasound has not been widespread.

In recent years, AI and machine learning-based ultrasound image analysis are gaining momentum as research subjects [4, 5, 10]. These technologies may offer a new advantage in improving outcomes and represent a training aid for operators that are not experienced in neuraxial insonation techniques [4].

Several applications of AI-US have been proposed: automatized identification of organ structures and lesions, assessment of disease status and specific categorization [11]. Two natural fields of implementation of neuraxial AI-US are obstetric and orthopaedic anesthesia. Automated landmark identification programs have been shown effective in identifying needle insertion points in obese pregnant women requiring spinal anaesthesia for cesarean Sect.  [5] as well as in epidural catheter positioning in parturients requesting labor epidural analgesia and in combined spinal–epidural anaesthesia for cesarean delivery, showing positive impact on increasing first-attempt success and shortening procedure's duration [4, 10].

When performing spinal anaesthesia in obese patients undergoing orthopaedic procedures, anesthesiologists needed to redirect the needle fewer times when AI-US was implemented. Of note, interspinous spaces identified as per digital palpation has been shown to be less precise when compared to AI-US; this inconsistency was also particularly evident in our case [12].

In conclusion, benefits brought to the field by AI-US are multiple, all reflected in significantly increased patient satisfaction. In both spinal and epidural anesthesia, AI-US increases efficacy of interspinous space location, reduces needle placement time and predicts needle direction for reaching of target structures as well their distances from skin [13, 14].

Neuroaxial s-US is an advanced skill that relies on the operator for providing accurate results.

When compared to s-US, AI-US provides the clinicians more detailed information that can be pivotal in more complex clinical scenarios. In Table 1 are summarized strengths and core features of both techniques.

**Table 1:** Strengths of different neuraxial ultrasound methods

Literature findings	s-US	AI-US
Immediate identification of anatomical structures Skin-to-epidural space distance Optimal entry point and angle for needle advancement	To be estimated	Automatically calculated
3D reconstruction	Not applicable	Provided by AI-implementation
Shortening of procedure time	No clear evidence	Proven in different studies
First-time pass success rate	Increased	Increased
What we have learnt from our case		
Applicability	Whole body	Selected structures
Operator-dependent method	High user dependency	Lower user dependency
Training requirement	Time consuming	Briefer specific training

There is still much room for improvement and we are far from considering AI-US the standard for neuraxial anaesthesia. When ultrasound became available for practical use at the bedside, it led to a change in our clinical practice, for instance, in the way we look at vascular access and at peripheral nerve blocks. This historical turning point came not smoothly. Clinical trials and accumulation of experience and expertise were needed to make practitioners accept the novelties. We do not know if AI-US will become the new paradigm in neuraxial ultrasound. However, we do think it is a powerful tool we must start considering in our algorithms as well as for further investigations, systematic studies on this subject are warranted.

## Data Availability

The data sets used and/or analysed during the current study are available from the corresponding author on request.

## Abbreviations

s-US: Standard ultrasound
AI-US: Artificial intelligence enhanced ultrasound
BMI: Body max index
